# Editorial: Applied data science in psychology

**DOI:** 10.3389/fpsyg.2024.1415036

**Published:** 2024-05-22

**Authors:** David Delgado-Gómez, Sara Jiménez-Fernández, Aaron Sujar, Jorge Lopez-Castroman

**Affiliations:** ^1^Department of Statistics, University Carlos III, Madrid, Spain; ^2^Department of Psychiatry, Virgen de las Nieves University Hospital, Granada, Spain; ^3^Department of Informatics and Statistics, Universidad Rey Juan Carlos, Móstoles, Spain; ^4^Department of Signal Theory and Communications, School of Engineering, University Carlos III of Madrid, Leganés, Spain

**Keywords:** psychometrics, validation, item response data, decision-making, methodology

The convergence of data science and psychology presents a promising frontier for research and innovation. We received 25 original contributions for this Research Topic, but only seven were deemed suitable for publication. This disparity highlights a potential gap in understanding the application of data science within psychology and underscores the need for further methodological development. Data science presents a versatile toolkit with diverse applications. We have identified its potential in validating psychometric scales, scrutinizing response integrity, and analyzing decision-making processes. Additionally, a new tool designed to enhance data science application has emerged.

Four of the studies were for validating relevant psychometric scales for the Chinese population. The first one validates a scale aimed at assessing childhood maltreatment in China, a pervasive issue with profound implications for both physical and mental wellbeing. Data compiled from 68 studies reveal alarming statistics in this country: 19.6% of children under the age of 18 years have experienced emotional abuse, 26.6% have experienced physical maltreatment, and 8.7% have experienced sexual abuse (Fang et al., 2015). Peng et al. evaluated the psychometric properties and normative data of the Childhood Trauma Questionnaire-Short Form (CTQ-SF) for Chinese adolescents in a nationally representative sample from diverse provinces. The most fitting model featured a four-factor structure, demonstrating robust internal consistency, test–retest reliability, and convergent validity.

Second, Meng et al. validated the Sussex Oxford Compassion for the Self Scale (SOCS-S) in a Chinese working sample of 1,132 participants. Self-compassion, rooted in Buddhist psychology, positively influences coping in daily life and organizational settings, promoting a positive self-concept. It has been associated to many positive outcomes including enhanced intrinsic motivation, reduced negative emotions, and improved adaptability. The study's significance is highlighted by a recent systematic review emphasizing the necessity of modifying existing instruments for compassion measurement to cater to diverse populations and cultures (Jiang et al., 2023). Employing classical test theory, item response theory (IRT), and network analysis, the research confirmed the SOCS-S's five-factor structure, with high internal consistency and measurement invariance across genders. IRT analysis revealed that all 20 items exhibited sufficient discrimination and acceptable difficulty indices among Chinese occupational groups.

Third, Tu et al. evaluated the psychometric properties of the 10-item Connor–Davidson Resilience Scale (CD-RISC-10) in a Chinese military personnel sample of 3,129 participants. The CD-RISC-10 is a widely used assessment of resilience. However, psychometric properties of CD-RISC-10 had not been investigated in the Chinese military personnel sample. The present study demonstrated that the CD-RISC-10 measurements in military personnel were reliable, valid, and consistent across military rank, gender, and time. In addition, the resilience score was strongly related to depression and anxiety symptoms, and it was predictive of military training performance.

Finally, the study by Lei et al. assessed the validity and reliability of the Chinese version of the Core Extrusion Schema-Revised (CES-R) for high school students. The core extrusion schema involves the belief that one's true self will be rejected, leading to a tendency to conceal one's authentic self, and it may be an important contributor to prevalent disorders such as the social anxiety disorder. Involving 1,334 students in Wuhan for item analysis and validation and 1,745 students in Zhejiang for questionnaire validation, the results indicated that the two-dimensional model of CES-R showed good fit, showing positive correlations with related measures. The Chinese CES-R demonstrated good validity and reliability for evaluating high school students, with acceptable measurement invariance across genders and grades.

Data science brings a multitude of uses and approaches concerning psychology. For instance, emerging technologies leveraging data science can verify the accuracy of responses.  Orrù et al. illustrated how individual responses to questions can be anticipated by estimating peer reactions to the same queries and how this anticipation can be leveraged to reconstruct an individual's response to a single item as well as the collective response to all items. In a cohort comprising 187 subjects, the use of machine learning models to reconstruct individual responses from peer estimations across two studies was validated: one focusing on anxiety-related inquiries and the other on the Dark Triad of personality traits. The findings indicated that individual responses to a single question could be predicted with 70–80% accuracy. Moreover, the overall participant-predicted score across all questions exhibited a correlation of 0.7–0.77 with actual results. The false consensus effect framework shows promise as a technique for reconstructing truthful responses, particularly in forensic settings where respondents are highly inclined to conceal their true responses and thus authentic test responses are unavailable.

Guenther and Lordan employed data science techniques to explore how the disposition effect varies across decision-making scenarios involving mean-reverting commodities and non-mean-reverting equities. Furthermore, they investigated whether a straightforward informational intervention highlighting the disposition effect could influence decision-making. This study conducted a within-subject experiment involving 193 professional traders. The findings indicated that, prior to the intervention, traders exhibited the disposition effect in a manner consistent with profit maximization objectives. Moreover, the study revealed that the informational intervention effectively altered the observed level of the disposition effect and subsequent decision-making. Notably, the simple informational intervention enhanced trader returns when deciding on non-mean-reverting securities, but conversely had adverse effects when applied to commodities. Their study highlights the power of simple interventions to make disproportionately large changes to decision-making.

Finally, data science can contribute to the development of practical tools. Lu introduces the metavcov package designed for multivariate meta-analysis (MMA). MMA is a powerful statistical technique that can provide more reliable and informative results than traditional univariate meta-analysis, which allows for comparisons across outcomes with increased statistical power. However, implementing appropriate statistical methods for MMA can be challenging. The metavcov package provides useful tools for conducting MMA with examples in R under a generalizable, statistically principled analytical framework. It is very flexible in accommodating functions for different effect sizes and functions for different coefficient estimation methods. It is very practical with functions for data visualization and handling missing values. As well as being statistically principled, it is helpful in practice that, once the model has been specified, multiple imputation can be conducted automatically.

## Author contributions

DD-G: Supervision, Writing—original draft, Writing—review & editing. SJ-F: Writing—original draft, Writing—review & editing. AS: Writing—original draft, Writing—review & editing. JL-C: Conceptualization, Supervision, Writing—original draft, Writing—review & editing.
